# Improving remote prescribing in a CAMHS community team during the COVID-19 pandemic

**DOI:** 10.1192/bjo.2021.191

**Published:** 2021-06-18

**Authors:** Sarah Tai, Hannah Chu-Han Huang, Oliver Batham, Brindha Anandakumar, Christopher Abbott

**Affiliations:** South London and Maudsley NHS Foundation Trust

## Abstract

**Aims:**

Prior to the COVID-19 pandemic, prescriptions were usually collected by patients/families in person from the CAMHS community team base. Due to social distancing measures introduced during the pandemic, face-to-face contact between staff and patients had to be minimised. This led to an increase in remote prescribing, including from home. Feedback from team doctors was that the process of following the Remote Prescribing Protocol (RPP) was taking up a significant portion of their day, preventing them from doing other clinical work.

Our aim was to reduce the time taken to complete a remote prescription to pre-pandemic levels (under 15 minutes).

**Method:**

We used PDSA methodology in this QI project:

1. Plan: Survey sent out to team duty doctors to identify the most time-consuming steps in RPP which could be safely delegated to administrative staff
2. Do: Email sent requesting administrative staff clarify several details with patients/families when they request a prescription. This included the names and doses of medication, how many days they had left, where they wanted the prescription sent to (home/pharmacy) and the relevant address. If the patient usually received their repeat prescription from their GP, they were re-directed to their GP
3. Study: Following the intervention above, team doctors recorded how long it took to complete a remote prescription

**Result:**

The average time taken to complete a prescription fell from 31 minutes (pre-intervention) to 22 minutes (post-intervention). The range of time taken also dropped from 10-241 minutes (pre-intervention) to 0-46 minutes (post-intervention). The medications taking above the average time to complete were more likely to be non-controlled drugs rather than controlled drugs (which one may typically think would be more time-consuming to write out).

**Conclusion:**

Whilst we have successfully reduced the time for remote prescribing, we have not reached the target of reducing it down to less than 15 minutes (pre-pandemic timings). As part of the next PDSA cycle, we have carried out a survey to ask what barriers remain. Checking patient's notes and recent prescriptions can still be inefficient. We propose introducing an intervention whereby this can also be safety delegated to administrative staff e.g. including a copy of the most recent prescription in the request.

In the future, we will continue to improve the RPP with further PDSA cycles and carry out an audit on the system on a regular basis to ensure standards are met.

